# The effect of neutral electrolyzed water as a disinfectant of eggshells artificially contaminated with *Listeria monocytogenes*

**DOI:** 10.1002/fsn3.1053

**Published:** 2019-06-14

**Authors:** Andres Rivera‐Garcia, Liliana Santos‐Ferro, Juan C. Ramirez‐Orejel, Lourdes T. Agredano‐Moreno, Luis F. Jimenez‐Garcia, David Paez‐Esquiliano, Eduardo Andrade‐Esquivel, Jose A. Cano‐Buendia

**Affiliations:** ^1^ Facultad de Medicina Veterinaria y Zootecnia, Department of Microbiology and Immunology Universidad Nacional Autónoma de Mexico (UNAM) Mexico City Mexico; ^2^ Facultad de Medicina Veterinaria y Zootecnia, Department of Animal Nutrition and Biochemistry Universidad Nacional Autónoma de México (UNAM) Mexico City Mexico; ^3^ Cell Nanobiology Laboratory, Department of Cell Biology. Faculty of Sciences Universidad Nacional Autónoma de México (UNAM) Mexico City Mexico; ^4^ Facultad de Medicina Veterinaria y Zootecnia, Department of Physiology and Pharmacology Universidad Nacional Autónoma de México (UNAM) Mexico City Mexico; ^5^ Departamento de Ingenieria Bioquímica Instituto Tecnologico de Celaya Guanajuato Mexico

**Keywords:** egg disinfection, electrolyzed water, *Listeria monocytogenes*

## Abstract

Neutral electrolyzed water (NEW) was tested as a disinfectant against *Listeria monocytogenes* on the surface of table eggs. Eggs were collected from a single Bovans White flock and were exposed to *L. monocytogenes*. Artificially contaminated eggs were divided into three different treatment groups: NEW, 2% citric acid solution (CAS), and saline solution (SS). To evaluate the bactericidal effect, the Mexican norm for antimicrobial activity determination protocol was performed. The observed bactericidal effect was compared against those obtained from CAS and SS. Bacterial cells present on the eggshells were quantified. NEW exhibited a significantly higher bactericidal effect than CAS when evaluated on the surfaces of chicken eggshells (6.11 log_10_CFU/ml reduction in vitro and a 2.18 log_10_ CFU/egg reduction on eggs vs. 1.06 log_10_CFU/ml in vitro reduction and 1.74 log_10_CFU/egg). Additionally, CAS was found to react with the carbonate egg shield, resulting in a loss of cuticle integrity. Mineral content of NEW‐treated eggshells was similar to SS‐treated eggshells; however, CAS‐treated eggshells showed a significant decrease in phosphorous concentration compared to NEW treatment. In this study, we demonstrated the effect of NEW and CAS on the integrity of the *L. monocytogenes* wall using transmission electron microscopy. To the best of our knowledge, this is the first report of the effect of NEW against *L. monocytogenes* on eggshells. Our results show that NEW is a viable alternative solution for the disinfection of table eggs that does not affect the cuticle or shell.

## 1 INTRODUCTION

Eggs constitute one of the most inexpensive sources of protein for humans. The Food and Agriculture Organization of the United Nations (2009) reported that eggs represent the principal food in developing countries. Eggshells provide protection against bacteria, but they can be contaminated with many different pathogens (Mine, Oberle, & Kassaify, 2003). In order to ensure the decrease of foodborne illnesses, different chemical solutions have been developed for cleaning and sanitizing eggs (Soljour, Assanta, Messier, & Boulianne, 2004; Wells, Coufal, Parker, & Mcdaniel, 2010; Zeweil, Rizk, Bekhet, & Ahmed, 2015). Some of these products do not have any effects against certain pathogens and they could be damaging different eggshell components like the cuticle or the shell itself, allowing the entrance of different types of bacteria (Mine et al., 2003; Wang & Slavik, 1998). The cuticle prevents bacterial penetration by covering pores on the eggshell which decreases shell permeability (Wang & Slavik, 1998). The washing process could eliminate this egg protection, and egg quality could be affected. In the United States, Australia, and Japan, table eggs are washed with detergents (Hutchison et al., 2004; Northcutt, Musgrove, & Jones, 2005) and then rinsed with a chlorine solution to reduce dirt, debris, and microbial load. The Mexican Department of Agriculture, Livestock, Rural Development, Fisheries and Food (National Service for Agroalimentary Public Health [SENASICA], 2011) recommended the use of 2% citric acid solution (CAS) for egg disinfection. The use of chlorine‐based sanitizers is very common worldwide. However, these disinfectants have some disadvantages, such as the removal of the eggshell cuticle and the generation of unhealthy by‐products (e.g., carcinogenic and mutagenic chlorinated compounds such as chloroform, trihalomethanes, chloramines, and haloacetic acids). These by‐products may have a carcinogenic effect or irritate workers' mucous membranes (Allende, McEvoy, Tao, & Luo, 2009; Bull et al., 2011; Gil, Selma, López‐Gálvez, & Allende, 2009; Legay, Rodriguez, Sérodes, & Levallois, 2010; Ohtsuka et al., 1997). Chlorine is corrosive and is included in the list of the Directive on Industrial Emissions (Integrated Pollution Prevention and Control [IPPC], 2007). Consequently, its use is banned in some European countries like Belgium, Denmark, Germany, and the Netherlands (Bilek & Turantaş, 2013; Fallik, 2014; Ölmez & Kretzschmar, 2009; Ramos, Miller, Brandão, Teixeira, & Silva, 2013). Although disinfection with chlorine is widespread in the fresh‐cut, meat, and poultry industries, there is worldwide interest in developing alternative disinfection strategies to minimize the environmental and public health impacts (Gopal, Coventry, Wan, Roginski, & Ajlouni, 2010; Meireles et al., 2014).

A viable alternative is the use of neutral electrolyzed water (NEW). It is a nonirritating solution and has been demonstrated to have lower cytotoxicity to mammalian cells than sodium hypochlorite (NaOCl; Wang et al., 2007). It is made by electrolyzing NaCl in water to generate hypochlorous acid (HOCl) and has a microbicide effect that is 80 times more effective than the hypochlorite ion (Kim, Hung, & Brackett, 2000). The electrolysis process creates different chlorine forms. The predominant species are hypochlorous acid (HOCl; 95%; Cheng, Dev, Bialka, & Demirci, 2012; Guentzel, Liang Lam, Callan, Emmons, & Dunham, 2008), hypochlorite ions, and trace amounts of chlorine (Cl_2_; Liao, Chen, & Xiao, 2007). These characteristics cause NEW to be less corrosive and have a longer shelf life than acidic EW (Rahman, Jin, & Oh, 2010). When NEW interacts with organic matter, it has been reported that it becomes water again (Huang, Hung, Hsu, Huang, & Hwang, 2008), which means it is environmentally friendly and causes no harm to humans (Al‐Haq, Sugiyama, & Isobe, 2005). The presence of HOCl is important because Cl_2_ can volatilize causing the bactericidal effect to be lost (Cui, Shang, Shi, Xin, & Cao, 2009). In mammals, HOCl is produced by neutrophils and macrophages through the oxidative burst pathway. It has microbicidal activity and reacts with thiol, thioether, various amino groups, nucleotides, and carbohydrates (Wang & Slavik, 1998).

The aim of this study was to evaluate NEW's bactericidal activity against *Listeria monocytogenes* which is a major pathogen linked to many of the largest outbreaks of foodborne bacterial enteritis (United States Department of Agriculture, 2014), and it can grow or survive at low temperatures, including 4°C (Luchansky et al., 2017). Egg recalls due to *L. monocytogenes* were announced in 2012 and 2014 (Paramithiotis, Drosinos, & Skandamis, 2017) even though there were no reports of outbreaks related to the consumption of those contaminated eggs. NEW's neutral pH, high oxidation–reduction potential (ORP), and environmentally friendly characteristics make it an alternative sanitizer for eggs that will not impact the eggs' physical properties or quality.

## 2 MATERIAL AND METHODS

### 2.1 Bacterial strain and inocula

The bacterial strain *L. monocytogenes* (ATCC 19115) was obtained from the American Type Culture Collection. The strain was confirmed by a Vitek 2 system (BioMérieux Cat. No. 27630) according to the manufacturer's instructions, and the bacterial cultures were maintained on Palcam agar (Neogen, Cat. No. 7669A). A single colony loop was placed in 200 ml of trypticase soy broth (TSB; Bioxon, Cat. No. 211670) and incubated overnight at 37°C in a shaker at 200 rpm (MaxQ6000, Cat. No. SHKE6000‐7, Thermo Scientific). The viable cell count was verified by serial dilution and the plate count/spread plate method as it was described by Boczek, Rice, and Johnson (2014).

### 2.2 Egg collection and allocation

Table eggs were obtained from the Center of Teaching, Research and Extension in Poultry Production at the Autonomous National University of Mexico. Eggs laid by 50‐week‐old Bovans White hens were visually inspected, and only intact eggs were included in further analyses. Eggs were disinfected using a hydrogen peroxide treatment to remove other bacteria and kept at 4°C, and at the time of all experiments, all eggs were 3 days old.

### 2.3 Strain preparation

A single colony of *L. monocytogenes* was grown in 50 ml of trypticase soy broth (TSB; BD Bioxon, Cat. No. 211670) at 37°C for 16 hr. Titration was conducted according to the Mexican Official Norm for aerobic plate counting (NOM‐092SSA1‐1994, 1994). Decimal serial dilutions were performed in PBS in a final volume of 10 ml. One hundred microliters of each dilution was plated on a petri dish containing 15 ml of trypticase soy agar (TSA) (MCDLAB, Cat. No. 7171). The plates were incubated overnight at 37°C, and plate counting was performed.

### 2.4 Preparation of manure slurry and inoculation

To simulate horizontal contamination, chicken manure was obtained from a private production center and was prepared as described by Bialka, Demirci, Knabel, Patterson, and Puri (2004) with some modifications. In brief, the manure was dried and sterilized at 121°C for 60 min. The chicken manure was weighed, and 200 g was then mixed with 2 L of sterile 0.1% peptone water. The bacteria were diluted in the chicken manure mixture to a concentration of 10^6^ CFU/ml. Selected eggs were allowed to reach room temperature and were divided into three groups (Figure S1). Each group was soaked in the chicken manure slurry for 10 min. Finally, the eggs were dried for 26 min in a laminar flow hood (Nuaire, Model NU‐440‐400, Cat. No. 503995).

### 2.5 Analysis of solutions

Neutral electrolyzed water was provided by Esteripharma Mexico S.A. de C.V. For these experiments, 2% CAS (Cat. No. 0110, J.T. Baker) and 0.9% saline solution (SS; NaCl, Cat. No. 6845) solutions were prepared, and a chemical evaluation was performed as follows: the pH and ORP were measured using a pH/ORP/temperature combo tester (Hanna, Cat. No. HI98121) following the manufacturer's instructions. The iodometric method was used (APHA/AWWA/WEF, 2012) to evaluate free chlorine content.

### 2.6 In vitro microbial challenge

According to the Mexican norm for antimicrobial activity determination (NMX‐BB‐040‐SCFI‐1999, 1999), we used a nonselective agar. The bacterial strain was obtained by following the strain preparation protocol described above. The *L. monocytogenes* strain was tested with NEW, CAS**,** and SS for 30 s. CAS and SS solutions were used as the disinfectant (positive control) and wash (negative control), respectively. Decimal serial dilutions were performed with 0.1% peptone water, and a 1‐ml aliquot of each dilution was plated on a petri dish containing TSA. The plates were incubated at 37°C overnight, and the colonies were counted. Plates containing 25–250 CFU were used to calculate titers. The percent reduction (*R*) was calculated using Equation 1.

(1) R(%)=100-((A∗100)/B)

where *A* is the number of viable microorganisms after treatment (CFU/ml), and *B* is the number of viable microorganisms before treatment or treated with SS (CFU/ml). All measurements were performed in triplicate.

### 2.7 Transmission electron microscopy

Microbial challenge samples were collected and pretreated as follows: bacterial samples from the in vitro microbial challenge were obtained, the samples were centrifuged (1,500 *g*) for 3 min, and the supernatant was discarded. The pellet was fixed with 2.5% glutaraldehyde–4% paraformaldehyde for 2 hr, rinsed three times with PBS for 5 min each, postfixed with 2% osmium tetraoxide, and rinsed with PBS. Subsequently, the preparations were dehydrated in graded concentrations of ethanol (from 30% to 100% for 5 min each) and propylene oxide. Finally, the samples were embedded in epoxy resin (Embed‐812) and cut into ultrathin sections (40‒60 nm) with an ultramicrotome (LEICA, EM UC7). The sections were mounted in formvar‐coated copper grids and double stained with uranyl acetate and lead citrate. Transmission electron microscopy (TEM) images were obtained using a TEM (JEOL 1200 EXII) with a camera (Gatan, Orius CCD) adapted to a microscope.

### 2.8 Treatment of contaminated shell eggs and microbiology analysis

Eggs exposed to *L. monocytogenes* were randomly divided into three groups (Figure S1) with 33 eggs per group. Eggs were allocated on open plastic egg trays. Eggs in the first group were treated with NEW, the second group was treated with CAS, and the third group was treated with SS as the wash‐control group. All treatments were performed using plastic spray bottles with 15 ml used per tray. Eggs were oriented vertically and disinfected with half of the treatment, and then, they were flipped upside down individually using sterile gloves and were treated with the rest of the volume. Treated eggs were incubated at room temperature for 1 min. The bacteria collection procedure was conducted as described by Fasenko, O'Dea Christopher, and McMullen (2009), with some modifications. Each egg was deposited in a plastic bag (Nasco Whirl‐Pak, B01065WA) containing 10 ml of 0.1% peptone water. Then, the eggs were rubbed by hand for 1 min, and 1 ml aliquots were taken from the plastic bags and used for plate counting.

### 2.9 Cuticle analysis

The evaluation of the integrity of the cuticle was conducted as described by Bialka et al. (2004) with some modifications. Eggs were contaminated as previously mentioned in the preparation of manure slurry and inoculation protocol and were divided into three groups with 14 eggs per group (Figure S1) and subjected to the disinfection treatments. After, eggs were submerged in 0.1% trypan blue (Merck, Cat. No. 111732) solution for 1 min, washed with water for 3 s, and dried in a laminar flow cabinet for 20 min. To quantify the color of each group, a spectrophotometer (Konica Minolta, CM‐600d) was used to measure the Lab color space (CIELAB), where *L* is the lightness (ranging from 0 [black] to 100 [white]), *a* reflects the axis from red to green, and *b* represents the axis from yellow to blue. Five random zones per egg were measured. Delta *E* was calculated using Equation 2.

(2) $$\Delta E=\sqrt{{\left(L_{2}-L_{1}\right)}^{2}+{\left(a_{2}-a_{1}\right)}^{2}+{\left(b_{2}-b_{1}\right)}^{2}}$$

where 1 is the value obtained from unstained eggs, and 2 is the value obtained from eggs stained with trypan blue.

### 2.10 Quantification of minerals in eggshells

Mineral quantifications were performed using the standard methods described in the Official Methods of Analysis of the Association of Official Analytical Chemists (AOAC, 1990). In brief, contaminated eggs were divided into three groups (Figure S1). Each group contained 14 eggs, and each group was treated as described previously. Treated eggs were stored at room temperature for 40 days. Subsequently, the amount of minerals (Ca, Mg, and P) in the eggshells were determined. The shells from seven eggs (in duplicate) were mixed and ashed at 550°C for 4 hr. Then, 15 ml of 3‐N HCl solution was added to the ashes. The obtained resuspension was filtered, and the volume was adjusted to 50 ml with deionized water. The phosphorus concentration was determined by ultraviolet (UV)–visible (VIS) spectrophotometry at 400 nm (Perkin Elmer, Lambda 2S) according to AOAC Official Method 965.17. The magnesium (285.2 nm) and calcium (422.7 nm) concentrations were measured using atomic absorption spectroscopy (Perkin Elmer, Model 3110) according to AOAC Official Method 968.08. For the mineral measurements, Sigma‐Aldrich standards of magnesium (Cat. No. 42992), phosphorus (Cat. No. 51474), and calcium (Cat. No. 69349) were used.

### 2.11 Statistical analysis

All statistical analysis was performed using the GraphPad Prism version 6.00 for Windows (GraphPad Software, www.graphpad.com) software. In all cases, probability was assessed at *p ≤ *0.05. Differences between nontreated and treated eggs were compared using a one‐way analysis of variance (ANOVA) at a 95% confidence level. For significant results, Tukey's multiple comparisons test was performed. Student's *t* test was used to compare obtained titers from Palcam and TSA plates.

## 3 RESULTS AND DISCUSSION

### 3.1 Physicochemical properties

All solutions were evaluated before use, and their properties are listed in Table 1. NEW properties (pH = 6.86; ORP = 872 mV; free chlorine concentration = 46 ppm) were similar to those reported for neutral electrolyzed solutions (Len, Hung, Erickson, & Kim, 2000). Other electrolyzed water (EW; Deza, Araujo, & Garrido, 2003) with similar ORP values (795‒816 mV) has been reported. The ORP is related to the presence of HOCl, and high ORP values are related to the disruption of the bacterial outer membrane and oxidation of intracellular reactions and respiratory pathways (Liao et al., 2007). This activity is related to changes in the electron flow inside the cells and the oxidation of some enzymes (Hati et al., 2012). SS solution showed a low ORP (375 mV) and a pH of 6.46 (near neutral). It has been reported that disinfectants with ORP values below 620 mV need to be in contact with *Listeria* spp. for more than 300 s (Suslow, 2004). The ORP of CAS was 623 mV, and the pH was 1.71. The bactericidal effect of CAS can be attributed to its low pH and to its ORP value (Arias‐Moliz, Ferrer‐Luque, Espigares‐Rodríguez, Liébana‐Ureña, & Espigares‐García, 2008). With all this data, NEW and SS showed a neutral pH, NEW had the highest ORP value and SS the lowest, and NEW was the only solution where free chlorine was detected.

**Table 1:** Properties of evaluated solutions^a^

	pH	ORP (mV)^b^	Cl (mg/L)^c^
NEW^d^	6.86 ± 0.1	872 ± 3	46 ± 1
CAS^e^	1.71 ± 0.1	623 ± 1	ND^g^
SS^f^	6.46 ± 0.1	375 ± 1	ND^g^

a Values represent the mean ± *SEM*, (*n* = 3).

b Oxidation reduction potential.

c Free chlorine.

d Neutral electrolyzed solution.

e 2% citric acid solution.

f 0.9% saline solution.

g Not detectable.

### 3.2 In vitro evaluation

**Figure 1:**
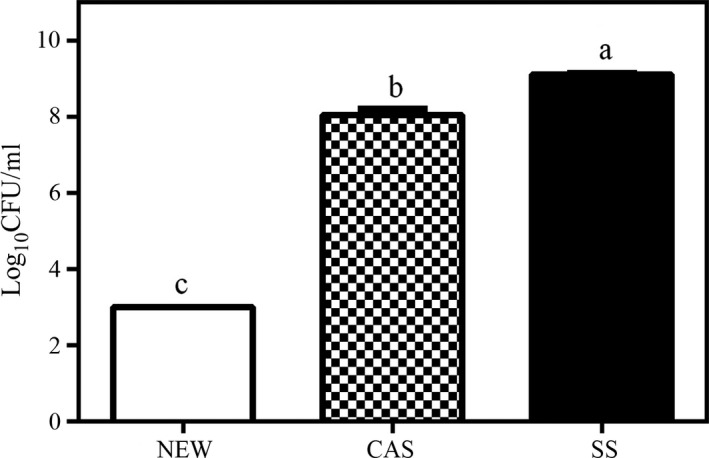
Effect of disinfectant and nondisinfectant solutions on *Listeria monocytogenes*. Bacteria were exposed to disinfectant neutral electrolyzed water (NEW), 2% citric acid solution (CAS), or nondisinfectant (SS) for 30 s and neutralized with peptone, and then, plate counting was conducted. Experiments were performed in triplicate. The values represent the mean ± *SEM* (log_10_CFU/ml). Different letters indicate significantly different means (*p* ≤ 0.01)

The bactericidal effects of NEW and CAS against *Listeria* were compared against the bacterial titers of the SS treatment. All solutions and *L. monocytogenes* were incubated together for 30 s. When the bacteria were treated with NEW, CAS, and SS, the titers were 3, 8.04 ± 0.1, and 9.1 ± 0.04 log_10_CFU/ml, respectively. The NEW treatment resulted in a 6.1 log_10_CFU/ml reduction from the original bacterial titer, corresponding to a bacterial reduction of >99.999%. CAS showed a reduction of 1.06 log_10_CFU/ml, which corresponds to a 91.03% decrease in the bacterial load (Figure 1). The differences (*p* < 0.01) between the three solutions were statistically significant.

Different types of EW have been evaluated (Kim et al., 2000; Russell, 2003; Venkitanarayanan, Ezeike, & Doyle, 1999) testing acidic EW (pH ˂ 2.7) with *Listeria* and reported a decrease in bacterial titers that ranged from 4 to 8.17 log CFU/ml. The biocidal activity of alkaline EW (pH 8.2; Deza et al., 2003) has been tested, and it has shown a decrease of 7.5 log CFU/ml in titers after 10 min of treatment. Another in vitro assay was reported (Liato, Labrie, & Aïder, 2017) using 1%, 3%, and 5% CAS. These solutions showed the lowest bactericidal effect against *L. monocytogenes, Staphylococcus aureus*, and* Staphylococcus enterica* in comparison with acetic and lactic acid.

### 3.3 Transmission electron microscopy

**Figure 2:**
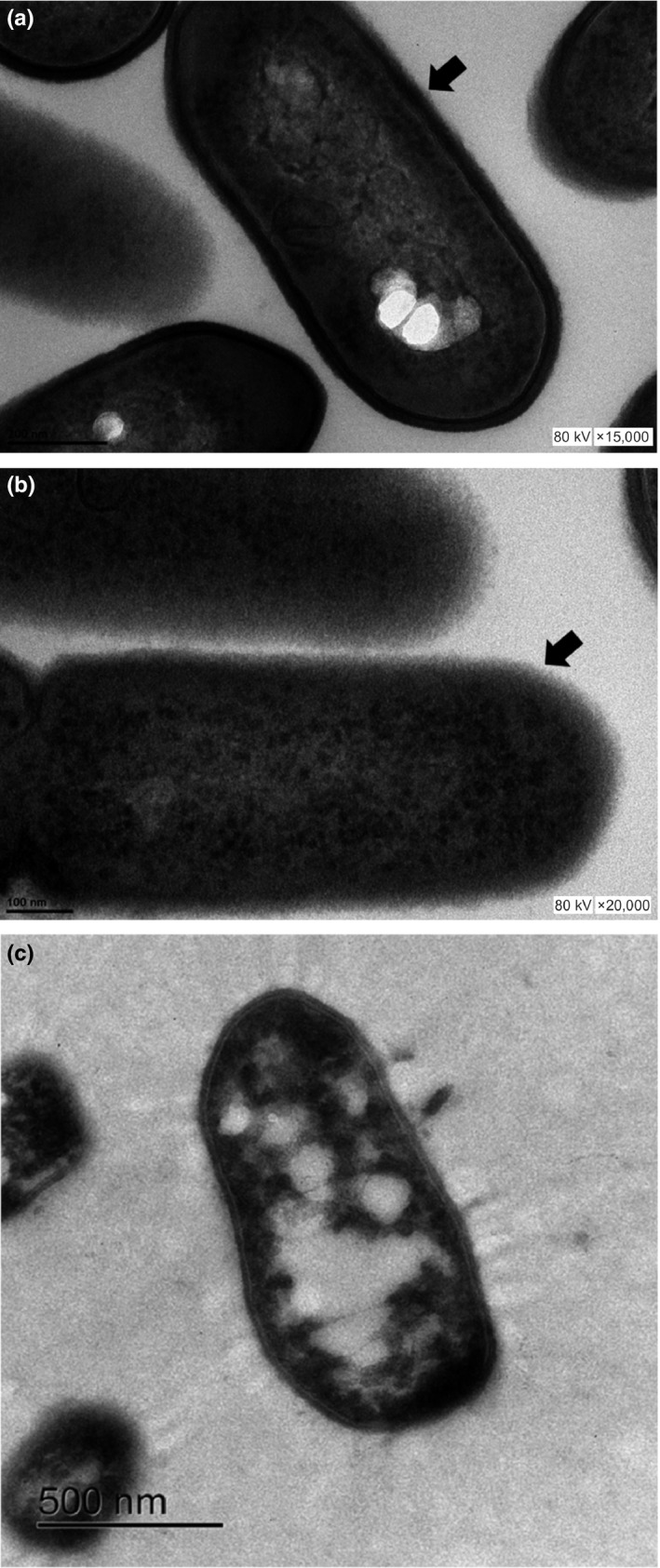
Transmission electron micrograph of *Listeria monocytogenes* ATCC 19115. Bacteria exposed to SS (a), NEW (b), or CAS (c) for 30 s. Control cells (SS) have an intact cell wall (black arrow). NEW‐treated bacteria exhibit a loss of integrity in the cell wall (black arrow). CAS caused cytoplasmic clumping and lack of cytoplasm

Many explanations for the action mechanisms of NEW have been described, including the inhibition of aldolase, disruption of protein synthesis and nucleic acids, generation of DNA damage, and inhibition of oxygen uptake and oxidative phosphorylation (Marriott & Gravani, 2006). One goal in our study was to determine whether or not damage to the bacterial surface occurred, especially since no pictures showing this type of damage exists in any of the previous reports where EW has been used. After treating *L. monocytogenes* with NEW and neutralizing the bactericidal effect, the samples were observed by TEM. Bacteria treated with SS solution showed normal morphology with clear and defined cell wall edges. However, when *Listeria* was treated with NEW for 30 s and then neutralized with peptone water, a loss of cell wall integrity was detected (Figure 2) as well as damage to the cell surface, the cell wall became undefined, and disorganization of cytoplasm (cytoplasmic clumping) was observed. Similar damage was described in *L. monocytogenes* by Saha et al. (2015) after heat treatment. In these images, NEW caused a disturbance in the cell wall. Liao et al. (2007) treated *Escherichia coli* with an electrolyzed oxidizing water (pH 2) and observed both internal and external damage to the bacterial membranes, which could cause bacterial death. As a disinfectant control, CAS was used, and we detected pore formation or the disruption of the cell membrane. We also detected cytoplasmic clumping and a lack of cytoplasm. CAS has a bactericidal effect because of its low pH (1.71). It passes through the membrane and acidifies the cytoplasm which causes the denaturation of organelles and bacterial death. These types of damage were reported previously where *Listeria innocua* (Feliciano, Lee, & Pascall, 2012) and *E. coli* (Liao et al., 2007) were used under different treatments.

### 3.4 Bactericidal effect on eggshells

The effects of disinfectants on the surfaces of eggshells exposed to *L. monocytogenes* were evaluated using the Mexican norm 040 (NMX‐BB‐040 ‐SCFI‐1999, 1999). This norm establishes the use of nonselective media.

**Figure 3:**
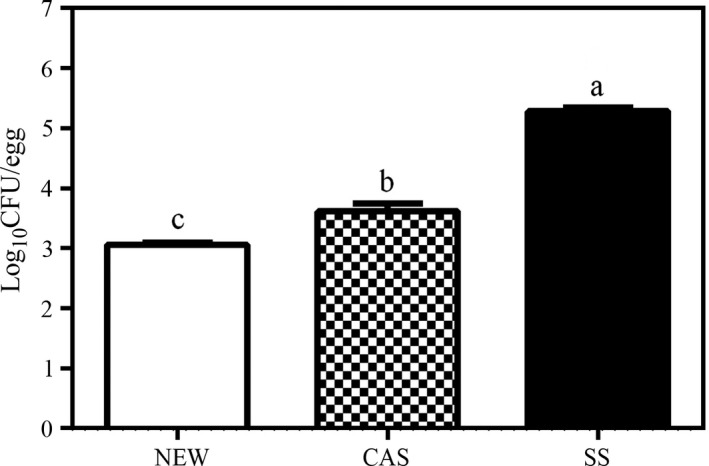
Effect on eggshells of disinfectant and nondisinfectant solutions on *Listeria monocytogenes.* Eggshells were exposed to *L. monocytogenes* and treated with 2% citric acid (CAS), neutral electrolyzed solution (NEW), or nondisinfection solution (wash‐control solution, SS) for 60 s. The bacterial survival rate was calculated (in triplicate). Values represent the means ± *SEM* (log_10_CFU/mL). Different letters indicate significantly different means (*p* ≤ 0.01)

Contaminated eggs with *L. monocytogenes* and treated with NEW showed a bacterial load of 3.06 log_10_CFU/egg, and 25 of the 33 treated samples did not have any bacterial grown on the plates; CAS group had a titer of 3.496 log_10_CFU/egg, and eight of 33 treated eggs did not show any growth too. SS group had a 5.24 log_10_CFU/egg titer, and bacterial growth was detected in all samples. These values correspond to reductions of 99.34% and 96.86% per egg when NEW or CAS treatments were applied, respectively (Figure 3). The differences were statistically significant (*p < *0.01) for both disinfectants analyzed. Previous works (Hannah et al., 2011; Mansour, Zayed, & Basha, 2015) reported similar titers and (Spitzer, 2015) reported that the main egg structure that is contaminated by bacteria is the eggshell and this contamination depends of the production method. We tried to increase the sensibility of the methodology from 1,000 to 100 CFU/egg by taking samples directly from the washing solutions (no‐dilution); however, we could not detect any bacteria in Palcam nor TSA plates (data not shown). However, the bacterial strains could have a survival rate below that limit.

The use of an acid EW on table eggs exposed to *L. monocytogenes* has been reported previously by Russell, 2003 with elimination rates ranging from 53% to 93%. The effectiveness of EW solutions for the elimination of pathogens other than *Listeria* has been evaluated on eggs in the past. Other research groups (Fasenko et al., 2009) have evaluated the use of EW in an egg washing process and reported a 1 log CFU/cm^2^ reduction in the natural aerobic bacteria of eggs. Ni, Cao, Zheng, Chen, and Li (2014) reported the use of EW with a pH = 5.74 against *Salmonella* Enteritidis, *E. coli,* and *S. aureus* which led to a reduction of 2.4, 2.71, and 2.78 log_10_CFU/g, respectively, in the bacterial population on the eggshells. Bialka et al. (2004) reported ˃2.6 log_10_CFU/g reduction rates against *E. coli* and *Salmonella* Enteritidis. Another group also reported the use of near neutral EW (pH 6.5) (Guentzel et al., 2008) with a reduction rate of 3 log CFU/mL, but it was evaluated on lettuce contaminated in vitro with *L. monocytogenes.*

Previously, the bactericidal effect of CAS against *L. monocytogenes* has been tested in a solution made of equal concentrations (0.8% final concentration) of citric acid, acetic acid, and propionic acid on chicken skin (Menconi et al., 2013). In that study, the reduction of the bacterial load on chicken skin was 1.85‒2.87 log_10_CFU. It is difficult to compare different reports because CAS was tested on foods like spinach (Finten, Agüero, & Jagus, 2017) with others components like glacial acetic acid (Yang, Kendall, Medeiros, & Sofos, 2009) or vinegar (Sengun & Karapinar, 2005). Moreover, another report (Maktabi, 2018) showed no effect of 1% CAS on eggs contaminated with *Listeria*. These studies demonstrated the antibacterial efficacy of EW against different pathogens, despite the varying physicochemical properties of the employed solutions. However, our research is the first report where a neutral EW has been used on eggs and statistically significant differences (*p* < 0.01) in the reduction of *Listeria* were observed between three groups: NEW, CAS, and SS.

### 3.5 Cuticle evaluation

The cuticle is a protein layer that covers the eggshell. To evaluate the presence of this layer, eggs were stained with trypan blue, and the resulting color was measured. After staining, the eggs were visually inspected. The CIELAB color space was used to quantify the observed differences in terms of the *L* (lightness), *a* (red/green), and *b* (yellow/blue) values. Before any treatment, eggs from different groups did not show any differences (*p* > 0.05) in *L*, *a*, or *b* values. After treatment and staining, all the values were statistically significantly different (*p* < 0.01). To obtain the global change, the Δ*E* value which includes *L, a*, and *b* values was calculated. A higher Δ*E* corresponds to major color changes. The CAS group produced higher numbers (Table 2). However, all differences were statistically significant (*p* < 0.01). Based on the Δ*E* values, the NEW group showed the smallest changes in color even though the eggs treated with CAS changed the most. This might be because trypan blue penetrated deeper into the eggshells where the reaction between the citric acid and eggshell carbonates likely led to a loss of cuticle and, as a result, exposed the pores to the trypan blue stain.

**Table 2:** Egg color related to cuticle***

Parameter	NEW^a^	CAS^b^	SS^c^
CIELAB *L* value^d^	58.34 ± 0.73^A^	45.26 ± 0.24^C^	52.85 ± 0.4^B^
CIELAB *a* value^e^	−3.07 ± 0.1^C^	−0.98 ± 0.13^A^	−2.31 ± 0.11^B^
CIELAB *b* value^f^	−28.95 ± 0.45^A^	−35.54 ± 0.12^C^	−32.02 ± 0.21^B^
Δ*E* g	48.23 ± 1.43^C^	61.36 ± 0.46^A^	53.5 ± 0.9^B^

a Eggs exposed to *Listeria monocytogenes* slurry and treated with neutral electrolyzed water.

b Eggs exposed to *L. monocytogenes* slurry and treated with 2% citric acid solution.

c Eggs exposed to *L. monocytogenes* slurry and treated with 0.9% saline solution.

d Lightness, ranging from 0 (black) to 100 (white).

e Ranging from red to green.

f Ranging from yellow to blue.

g Global change in color.

*** Values represent the means ± *SEM* within a row without a common superscript are significantly different (*p* < 0.001).

### 3.6 Quantification of minerals in eggshell

Results from the cuticle evaluation suggested that CAS treatment affects cuticle integrity. Calcium, magnesium, and phosphorous concentrations were determined to evaluate whether or not CAS treatment affected the eggshell. There were no significant differences (*p* > 0.05) between mineral values from eggs treated with SS and NEW solutions. Phosphorous concentration in CAS‐treated eggs was lower compared to those found in eggs treated with NEW (Table 3). Cusack, Fraser, and Stachel (2003) reported that the amounts of magnesium and phosphorus in eggshells decrease significantly when the integrity of the cuticle is compromised, and they are the major minerals that constitute the eggshell. They are concentrated on the eggshell surface (Cusack et al., 2003). Phosphorus is present as phosphoproteins in the cuticle (Gautron, Hincke, & Nys, 1997). Therefore, changes in phosphorus concentrations could be related to cuticle damage allowing for entrance of bacteria or the colonization of cuticle‐digesting bacteria like *Pseudomonas*, producing spoiled eggs and affecting the shelf life or internal quality (Rodríguez‐Navarro, Domínguez‐Gasca, Muñoz, & Ortega‐Huertas, 2013). CAS treatment caused a low concentration of magnesium and phosphorous on treated shells, and this effect could allow to treated eggs be more susceptible to infection because the integrity is compromised.

**Table 3:** Eggshell mineral contents**

Mineral	NEW^a^	CAS^b^	SS^c^
Ca (mg/g)	191.46 ± 13.42^A^	194.89 ± 8.8^A^	227.32 ± 8.25^A^
Mg (ppm)	6,692.45 ± 743.4^A^	5,536.35 ± 505.8^A^	6,101.61 ± 592^A^
P (ppm)	647.86 ± 54.42^A^	473.86 ± 33.83^B^	573.51 ± 18.97^AB^

a Eggs exposed to *Listeria monocytogenes* slurry and treated with neutral electrolyzed water.

b Eggs exposed to *L. monocytogenes* slurry and treated with 2% citric acid solution.

c Eggs exposed to *L. monocytogenes* slurry and treated with 0.9% saline solution.

** Values represent the mean ± *SEM*. Means within a row without a common superscript are significantly different (*p* ≤ 0.01).

In vitro experiments revealed that NEW exerts a bactericidal effect on chicken eggshells. Eggs are laid on shavings or on slats; it has been reported that nonwashed and washed eggs contain bacterial loads (Hannah et al., 2011; Mansour et al., 2015). Hannah et al. (2011) reported bacterial titers on nonwashed eggs from 0.6 to 4.2 Log_10_CFU/ml and on washed eggs from 0.2 to 2.5 Log_10_CFU/ml of eggshell rinsates. Strain identification revealed *E. coli* and some other species, but neither of those works looked for *Listeria*. However, in our study we used higher amounts of bacteria (inoculum: 10^6^ CFU/ml), resulting in an *L. monocytogenes* load of 10^5^ CFU/egg, and this load was depleted below our detection limits. Hannah et al. (2011) and Mansour et al. (2015) reported a prevalence of 68% for aerobic bacteria at loads of ~2.3 log_10_CFU/ml in eggshell rinsate. This model revealed that NEW could reduce bacterial load without affecting the cuticle or the mineral composition of the eggshell. The maintenance of the shell integrity could be attributable to the lack of reaction between NEW and the cuticle after the disinfection process. Our results revealed no decrease in the mineral contents of chicken eggshells, suggesting that this process does not affect the eggs' resistant characteristics.

## 4 CONCLUSION

In summary, the results from our study support that NEW is a good candidate to use as a sanitizing solution to reduce/eliminate *L. monocytogenes* from the shell of table eggs without affecting the integrity of the cuticle or the eggshell's mineral content. It also does not change the color of the eggshell after its use.

## CONFLICTS OF INTEREST

The authors declare no conflict of interest.

## ETHICAL STATEMENT

This study does not involve any human or mammal testing.
