# Supplementary figures and images for: The effect of neutral electrolyzed water as a disinfectant of eggshells artificially contaminated with Listeria monocytogenes
Source: Food Sci Nutr. 2019 Jun 14;7(7):2252–60. doi: 10.1002/fsn3.1053 (PMC6657710; doi:10.1002/fsn3.1053)

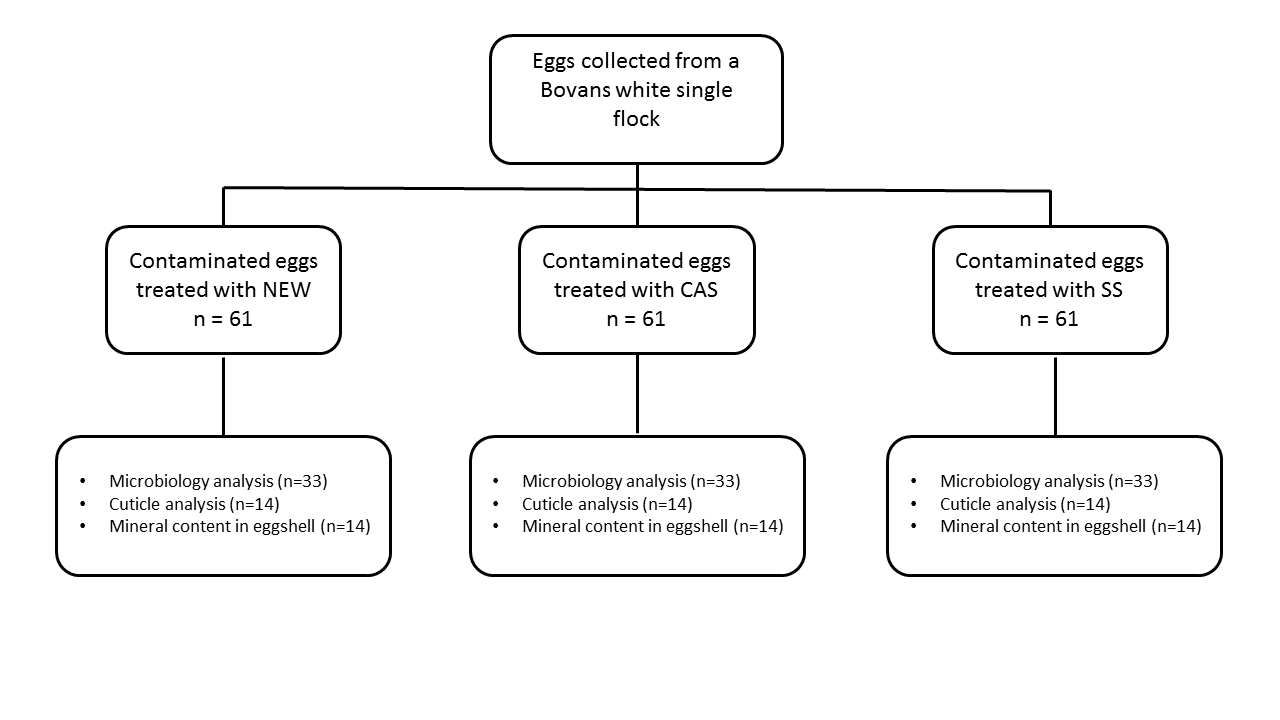

Supplement: Supplementary file 1 [file FSN3-7-2252-s001.tif]
