# Supplementary material for: The effect of neutral electrolyzed water as a disinfectant of eggshells artificially contaminated with Listeria monocytogenes
Source: Food Sci Nutr. 2019 Jun 14;7(7):2252–60. doi: 10.1002/fsn3.1053 (PMC6657710; doi:10.1002/fsn3.1053)
Supplement: Supplementary file 2 [file FSN3-7-2252-s002.docx]

**Figure 1 Supp**. Flowchart of the allocation of eggs to three treatment groups. All eggs were collected from a single Bovans White flock. Each group was exposed to a *L. monocytogenes* slurry and washed (treated) with different solutions (0.9% NaCl solution, SS; 2% citric acid solution, CAS; neutral electrolyzed solution, NEW). Eggs were subjected to enlisted analysis.
